# A Strategy for Fabricating Ultra-Flexible Thermoelectric Films Using Ag_2_Se-Based Ink

**DOI:** 10.3390/ma17153784

**Published:** 2024-08-01

**Authors:** Yunhuan Yuan, Chaogang Ding, Rui Yin, Shun Lu, Jie Xu, Wei Ren, Kang Li, Weiwei Zhao

**Affiliations:** 1 Flexible Printed Electronics Technology Center, Harbin Institute of Technology, Shenzhen 518055, China; yuanyunhuan@163.com (Y.Y.); yinrui37@163.com (R.Y.); wzhao@hit.edu.cn (W.Z.); 2 Key Laboratory of Micro-Systems and Micro-Structures Manufacturing of Ministry of Education, Harbin Institute of Technology, Harbin 150001, China; dingcg@hit.edu.cn (C.D.); xjhit@hit.edu.cn (J.X.); 3 Chongqing Institute of Green and Intelligent Technology, Chinese Academy of Sciences, Chongqing 400714, China; ysfield@live.com; 4 Changsha Semiconductor Technology and Application Innovation Research Institute, College of Semiconductors, Hunan University, Changsha 410082, China

**Keywords:** flexible thermoelectric materials, printable ink, screen printing, low-temperature sintering, printed film

## Abstract

Flexible thermoelectric materials have drawn significant attention from researchers due to their potential applications in wearable electronics and the Internet of Things. Despite many reports on these materials, it remains a significant challenge to develop cost-effective methods for large-scale, patterned fabrication of materials that exhibit both excellent thermoelectric performance and remarkable flexibility. In this study, we have developed an Ag_2_Se-based ink with excellent printability that can be used to fabricate flexible thermoelectric films by screen printing and low-temperature sintering. The printed films exhibit a Seebeck coefficient of −161 μV/K and a power factor of 3250.9 μW/m·K^2^ at 400 K. Moreover, the films demonstrate remarkable flexibility, showing minimal changes in resistance after being bent 5000 times at a radius of 5 mm. Overall, this research offers a new opportunity for the large-scale patterned production of flexible thermoelectric films.

## 1. Introduction

Thermoelectric materials offer a distinctive capability of directly converting a small amount of heat into electrical power, offering a reliable power solution that can potentially mitigate energy deficits [1,2,3]. The emergence of flexible thermoelectric materials has been particularly enticing for their potential applications in wearable electronics and the Internet of Things (IoT), as they can function as power sources for conformable electronic devices. Consequently, the quest for advancing flexible thermoelectric materials has become a prominent research domain in the energy sector in recent times [4].

However, the majority of conventional thermoelectric materials exhibit a rigid and brittle nature, impeding their suitability for the complex geometric curvatures of the human body and the demands of wearable electronics [5,6]. As a result, myriad materials, such as conductive polymers, inorganic semiconductors, metals, and carbon materials, have been explored for the development of flexible thermoelectric films over the past decade [6,7,8,9,10]. Among these, inorganic semiconductors display superior thermoelectric properties but suffer from inadequate flexibility [11]. On the contrary, conductive polymers offer substantial flexibility, yet their thermoelectric properties are suboptimal [12]. Presently, research efforts predominantly focus on enhancing flexibility while preserving the thermoelectric performance of these materials. Numerous attempts have been made to develop conductive polymer/inorganic semiconductor composites to achieve superior and flexible thermoelectric films, but the outcomes are often unsatisfactory [12,13,14]. For instance, the flexible PEDOT/Bi_2_Te_3_ composite film developed by Wang et al. exhibited promising results, but the involved process was complex, and the material’s flexibility requires further enhancement [15]. Furthermore, the limited abundance of the Te element on Earth presents additional challenges.

Silver selenide, due to its impressive power factor and low thermal conductivity at ambient conditions, emerges as a potential substitute for commercial Bi_2_Te_3_-based alloys [16,17,18]. There have been endeavors to fabricate flexible thermoelectric films employing silver selenide-based materials. For instance, a filtration method was used by researchers to create Ag_2_Se or its composite material films on nylon [19,20]. Though these films displayed good thermoelectric performance and flexibility, their flexibility could be further improved. Furthermore, the filtration method poses challenges for patterning and mass production.

Screen printing technology, owing to its ease of processability and high throughput, has gained significant interest in fabricating flexible thermoelectric films with desired dimensions and shapes [21]. However, the resultant thermoelectric materials often exhibit inferior printability or compromised electrical transport properties post-printing, necessitating further research to improve printability while retaining their intrinsic properties [22]. Specifically, there are limited studies reporting the fabrication of flexible thermoelectrics with superior performance using silver selenide-based materials for printing. Although Mallick et al. generated Ag−Se-based thermoelectrics via screen printing, the flexibility was not studied in detail, and the power factor could be enhanced [23]. The researchers also printed thermoelectric materials with excellent power factors, but these samples were inflexible [24]. Additionally, another group screen-printed silver selenide/PVP to achieve thermoelectric films with some flexibility, but the power factor was a mere 4.3 μW/m·K^2^ [25]. Thus, developing an Ag_2_Se-based material that exhibits optimal printability and high performance remains a formidable challenge. Additionally, the resultant thermoelectrics should demonstrate superior flexibility to cater to the needs of wearable electronics and IoT.

This study presents the development of a novel Ag_2_Se-based ink with commendable printability, enabling the fabrication of thermoelectric films with outstanding flexible and thermoelectric properties through screen printing and low-temperature sintering. Notably, the film displays a Seebeck coefficient of −145.4 μV/K at room temperature and a power factor reaching 1075.8 μW/m·K^2^. At 400 K, the Seebeck coefficient is −161 μV/K, and the power factor reaches 3250.9 μW/m·K^2^. Additionally, this film showcases superior mechanical flexibility, with its resistance remaining virtually unchanged after 5000 bending cycles at various bending radii. Collectively, this strategy meets the prerequisites for wearable electronics and IoT by facilitating the mass production of ultra-flexible thermoelectric films utilizing inexpensive and abundant elements.

## 2. Materials and Methods

### 2.1. Experimental Materials

AgNO_3_ (99.85%) was purchased from Innochem (Beijing, China). L-ascorbic acid (99.99%), β-cyclodextrin (98%), and SeO_2_ (99%) were purchased from Macklin (Shanghai, China). Chlorine vinegar resin (SOLBIN A) was obtained from Nissin Chemical Industry (Fukui, Japan). Polyester polyol was obtained from Zuo Rong Gong Industrial (Dongguan, China). Cyanate ester (SBN-70D) was purchased from AsahiKASEI (Tokyo, Japan). Isophorone was purchased from Aladdin (Shanghai, China).

### 2.2. Preparation of Printing Ink and Film

The synthesis of Ag_2_Se nanowires is a modification of the synthesis method proposed by Jeong et al. [26]. To fabricate Ag_2_Se nanowires, we initially obtained selenium nanowires (Se NWs) using the method detailed below. In a standard procedure, 1 g of SeO_2_ and 1 g of β-cyclodextrin were combined in a conical flask filled with 200 mL of ultrapure water and stirred for 20 min to create solution A. Concurrently, we dissolved 1.5 g of pure ascorbic acid in a separate flask containing 200 mL of ultrapure water and stirred vigorously to form solution B. Solution A was then gradually added to solution B while stirring continuously. After a period of five hours, the precipitate was obtained through repeated centrifuging and rinsing with ultrapure water and ethanol. Finally, we produced the selenium nanowires by letting them rest undisturbed in absolute ethanol for 48 h. The recovered Se NWs were then spun down and dried in preparation for the Ag_2_Se nanowire production. For the creation of Ag_2_Se nanowires, 0.32 g of Se NWs were redispersed in 320 mL of ethylene glycol (EG). We then separately dissolved 1.5 g of AgNO_3_ and 4.5 g of ascorbic acid in 15 mL of ultrapure water, applying ultrasonic treatment for 5 min each. Following this, we added the AgNO_3_ solution to the Se NWs solution while stirring vigorously. Afterwards, the ascorbic acid solution was gradually introduced into the resulting mixture. After 6 h of continuous stirring, we obtained a dark gray precipitate by centrifuging and washing multiple times with ultrapure water and ethanol. We finally obtained Ag_2_Se nanowire powder by drying it in a 40 °C vacuum oven.

The Ag_2_Se ink was formulated by combining 55 wt% Ag_2_Se nanowire powder, 7.5 wt% chloroacetic resin, 3 wt% polyester polyol, 8.5 wt% cyanate ester, and 26 wt% isophorone solvent. We then thoroughly mixed these components under a gravity mixer to achieve a homogenous ink. Subsequently, Ag_2_Se ink was screen-printed onto the copy paper to obtain the film, which was then placed in a 140 °C oven for one hour for sintering.

### 2.3. Materials Characterization

Scanning electron microscopy (SEM) images were captured using a scanning electron microscope (Carl Zeiss AG, Oberkochen, Germany). Transmission electron microscopy (TEM) and high-resolution TEM (HRTEM) images were recorded by a high-resolution transmission electron microscopy (JEM-2100, JEOL, Tokyo, Japan). X-ray diffraction (XRD) patterns were measured using a D/max 2500PC. Elemental analyses were determined by a Thermo Fisher Scientific K-Alpha (Thermo Fisher Scientific, Tokyo, Japan). The cross-section of a film was acquired with an ion beam slope cutter (Leica EM TIC 3X, Leica, Wetzlar, Germany) or with a scalpel under liquid nitrogen conditions. Digital image correlation (DIC) photographs were acquired by an Olympus microscope (SZX16, Tokyo, Japan). The resistance of samples was measured using a multifunction digital four-point probe tester (ST-2258C, Suzhou, China) or multimeter (DAQ6510, Cleveland, OH, USA). The carrier transport performance was determined by the standard van der Pauw technique (Quantum Design, San Diego, CA, USA). The thermoelectric performance, including electrical conductivity (σ) and Seebeck coefficient (S), was measured using PPMS-9, THERMAL TRANSPORT OPTION. 

## 3. Results and Discussion

### 3.1. Characterization of Obtained Powders and Films

**Figure 1:**
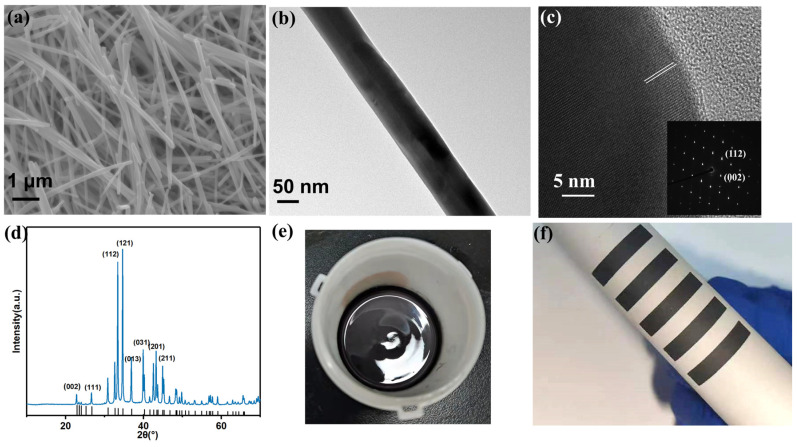
Characterization of these samples. (**a**) Typical SEM, (**b**) TEM, (**c**) HRTEM images (the inset is the SAED pattern recorded from this nanowire), and (**d**) XRD of the Ag_2_Se NWs. (**e**) Ink based on Ag_2_Se NWs. (**f**) The printed film.

First, the morphology and elemental composition of Ag_2_Se nanowires obtained are characterized using scanning electron microscopy (SEM), transmission electron microscopy (TEM) techniques, X-ray diffraction (XRD), and X-ray photoelectron spectroscopy (XPS) measurements. To synthesize Ag_2_Se, Se nanowires are utilized as templates, with ethylene glycol serving as both a solvent and a reducing agent. Ascorbic acid is employed to boost the reduction effect. The Ag^+^ cations undergo reduction to Ag atoms, which subsequently react with the Se nanowires, leading to the formation of Ag_2_Se nanowires [26]. Figure S1 shows the basic morphology of the precursor of Ag_2_Se (Se nanowires), with a length of approximately 10 μm. The X-ray diffraction (XRD) pattern matches well with the selenium standard card (PDF#06-0362). Furthermore, SEM and TEM images of Ag_2_Se NWs reveal that the lengths of Ag_2_Se NWs range from a few micrometers to several tens of micrometers, and the distribution of NWs is uniform (Figure 1a,b). HRTEM images of Ag_2_Se NWs show that the lattice spacing is 0.39 nm, which can be attributed to (002) crystal planes (Figure 1c). The inset of Figure 1c shows the selected area electron diffraction (SAED) pattern, with diffraction spots indexed as (002) and (112) of orthorhombic β-Ag_2_Se. From the morphology and SAED pattern, it can be inferred that the nanowire is oriented in the [002] direction. 

Furthermore, the XRD spectrum is consistent with the standard card (PDF# 24-1041) (Figure 1d). Among them, the peak at 45.2° can be attributed to the crystal plane (211). The strong peaks at 33.5° and 34.6° belong to the (112) and (121) crystal planes of the standard XRD card, respectively. As for the elemental composition of the Ag_2_Se sample, the peaks appearing at 368.4 eV and 374.4 eV were assigned to Ag 3d (Figure S2a), while the binding energies around 54.5 eV can be ascribed to Se 3d (Figure S2b) [19,27]. In summary, by analyzing the sample morphology, crystal structure, and chemical composition, it can be determined that the material obtained is Ag_2_Se nanowires.

**Figure 2:**
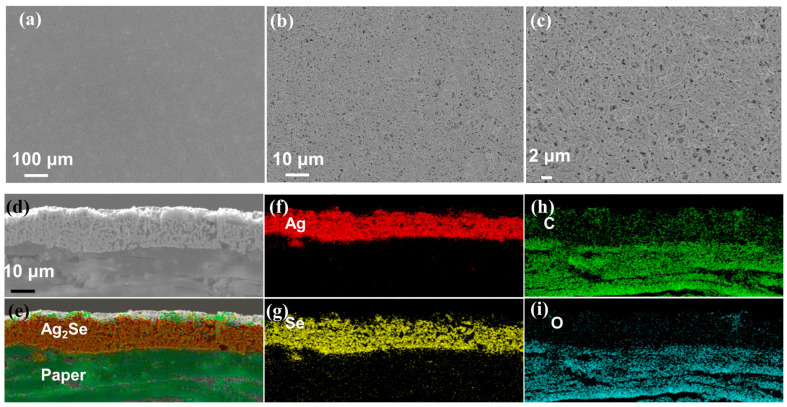
Surface and cross-sectional microstructure of the printed films after sintering. (**a**,**b**) Low magnification of the SEM surface images. (**c**) High magnification of the SEM surface images. (**d**) The cross-sectional image of the film. (**e**) The overall corresponding cross-sectional EDS image. (**f**) Ag, (**g**) Se, (**h**) C, and (**i**) O EDS images.

After acquiring the powder of Ag_2_Se nanowires, it is mixed with chloroacetic resin, polyester polyol, cyanate ester, and isophorone solvent in specific proportions. The mixture is then thoroughly stirred to achieve a uniform ink, which is used to fabricate flexible Ag_2_Se films (Figure 1e,f). Figure 2 shows the surface and cross-sectional morphology of the screen-printed film after sintering. As depicted in Figure 2a–c, the film surface is remarkably smooth, and the nanowires are clearly fused together, demonstrating high density. Resin is typically used as a binder to prepare ink [28]. Both vinyl chloride resin and polyester polyol are excellent binders. Moreover, when sintered at lower temperatures, they do not completely evaporate, thereby increasing the density of the film [29]. These all contribute to the tight binding of nanowires, thereby improving the electrical performance and flexibility of the film [28,29]. The Ag_2_Se nanowires have a diameter of only a few tens of nanometers, resulting in a large surface area; thus, they can be sintered at lower temperatures to form dense films without damaging the paper substrate [30,31]. The tight binding of these nanowires is beneficial for the performance of the material’s intrinsic properties, ensuring that the film does not interfere with electron transmission due to large voids, which could otherwise affect conductivity [29]. It is worth noting that typically, screen-printed TE-thick films exhibit numerous pores and porous interfaces due to the evaporation of solvents and binders during the high-temperature sintering process. Consequently, the film density is relatively low, resulting in higher electrical resistance [21,22,29]. 

The film is processed by an ion beam slope cutter for the investigation of its sectional microstructure. Subsequently, SEM and EDS are employed to obtain the detailed morphology and elemental composition of the cross-section. As shown in Figure 2c from SEM images, the overall film thickness measures approximately 120.33 ± 4.93 μm, with the upper film thickness being 16.95 ± 1.45 μm. These EDS images of heterointerface demonstrate that this film mainly contains Ag, Se, C, and O, with a small amount of Ag and Se penetrating the substrate during printing. The lower layers comprising C and O can be attributed to the paper, whereas the upper layers predominantly originate from the unevaporated resin. Due to the hot sintering process and the binder, the upper Ag_2_Se nanowires are well combined with the paper substrate, resulting in a composite film that exhibits excellent flexibility [31,32].

### 3.2. Electrical Transport Properties

**Figure 3:**
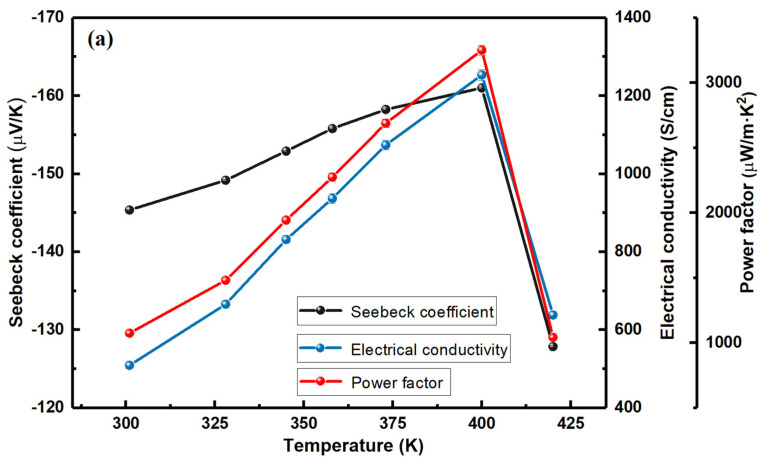

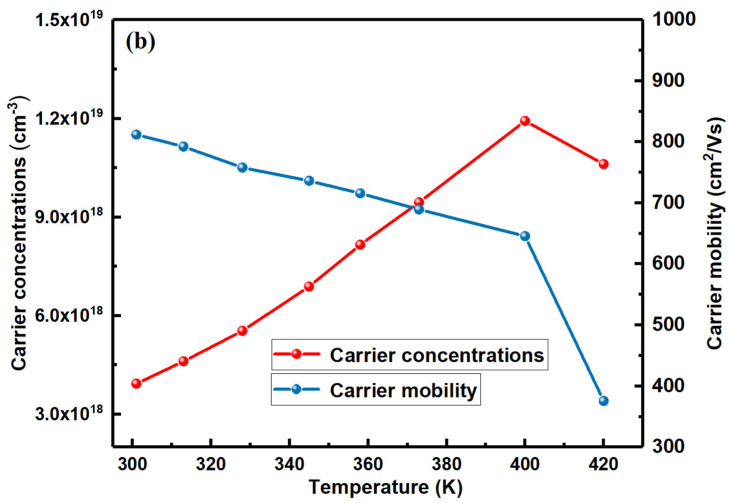
Electrical transport properties of the printed film. (**a**) The Seebeck coefficient, electrical conductivity, and power factor of the film with a temperature varying from 300 K to 420 K (**b**) The carrier concentration and mobility of the film with a temperature varying from 300 K to 420 K.

Figure 3 illustrates the electrical transport properties of the screen-printed Ag_2_Se film over a temperature range of 300 K to 420 K. The Seebeck coefficient (S) and electrical conductivity (σ) are measured as temperature variables. Figure 3a displays the average values (each point is tested 20 times using a PPMS-9). The Seebeck coefficient at room temperature is −145.4 ± 0.06 μV/K, and it gradually increases as the temperature rises from 300 to 400 K. However, as the temperature continues to rise from 400 to 420 K, the absolute Seebeck coefficient experiences a significant drop from approximately −161 ± 0.14 μV/K to −127.9 ± 0.1 μV/K. The trend of the Seebeck coefficient variation is similar to that of the Ag_2_Se film prepared by magnetron sputtering [33]. 

The conductivity of the film at room temperature is 509.1 ± 7.9 S/cm, which increases drastically with a temperature increase from 300 K to 400 K. However, when the temperature increases to 420 K, the conductivity drops dramatically from 1253.8 ± 13.9 to 638.2 ± 9.5 S/cm. The above phenomenon can be explained by the phase transition of Ag_2_Se from the orthorhombic semiconducting β-phase to the cubic superionic α-phase at 407 K. According to the literature, this process is generally reversible [34,35]. Among them, β-Ag_2_Se typically has high conductivity, a moderate Seebeck coefficient, and a high Hall mobility [33,36]. Likewise, the power factor (PF) at room temperature stands at 1075.8 ± 16.9 μW/m·K^2^, exhibiting a significant increase as the temperature rises, peaking at 3250.9 ± 38.9 μW/m·K^2^ at 400 K. However, at 420 K, the power factor experiences a steep decline, decreasing to 1043.5 ± 15.5 μW/m·K^2^. Compared to previous reports using screen-printed silver selenide films [23], which achieved a maximum power factor of 500 μW/m·K^2^, the thermoelectric performance of our silver selenide films has been improved. This advancement is beneficial for promoting the large-scale production of flexible thermoelectric devices based on silver selenide using screen printing.

The carrier concentration dramatically rises with temperature, increasing from 3.9 × 10^18^ at 300 K to 1.2 × 10^19^ at 400 K (Figure 3b). After the phase transition temperature point, it drops down to 1.1 × 10^19^ cm^−3^. The carrier mobility decreases slowly from 811.9 to 645.7 cm^2^/Vs within the temperature range of 300 K to 400 K, and a rapid decline is observed when the temperature reaches 420 K. This trend is also caused by the phase transition. Specifically, compared with a static lattice, the Ag ions in the superionic Ag_2_Se tend to move freely and scatter electrons more effectively [36].

### 3.3. Highly-Flexible Properties of the Screen-Printed Ag_2_Se Film

**Figure 4:**
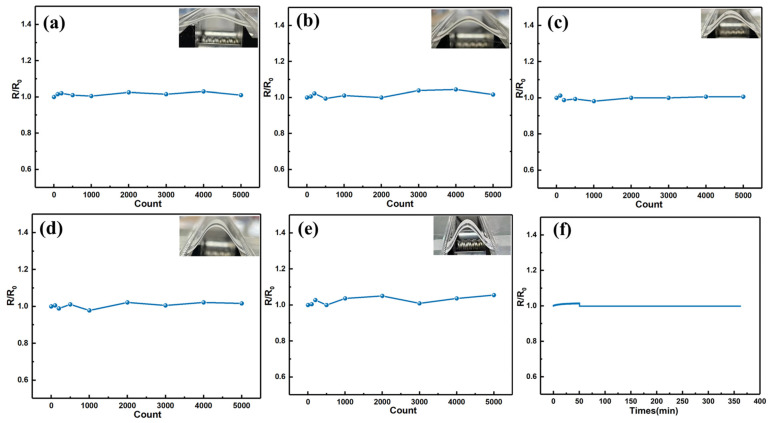
The variation of the resistance ratio (R/R_0_) with the change in bending radius and bending cycle. (**a**) Variation of R/R_0_ for a bending radius of 15 mm. (**b**) Variation of R/R0 for a bending radius of 11 mm. (**c**) Variation of R/R0 for a bending radius of 9 mm. (**d**) Variation of R/R_0_ for a bending radius of 7 mm. (**e**) Variation of R/R_0_ for a bending radius of 5 mm. (**f**) The real-time R/R_0_ variation curve of the sample fixed on an automatic bender with a 5 mm bending radius during 5000 bending cycles.

Traditional inorganic thermoelectric materials, such as Bi_2_Te_3_, GeTe, and PbTe, typically have poor mechanical properties, which can potentially lead to equipment failures [5,37]. To test the mechanical flexibility of these screen-printed films, the films are fastened parallel to an automatic bending machine using rulers and clamps and then repeatedly bent to the specified minimum radius. The test was conducted at a temperature of 25 °C. The ratio (R/R_0_) of the electrical resistance is measured as a function of the bending radius and bending cycles (Figure 4). When the bending radius is set at 15 mm, 11 mm, 9 mm, and 7 mm, the resistance of the film largely remains consistent even after 5000 bends (Figure 4), suggesting that the mechanical damage inflicted on the sample is minimal. To further investigate the flexibility of the film, the bending radius is reduced to 5 mm. After 1000 bends, the resistance only rises to 1.04 times the initial value. As the bending is extended to 5000 times, the resistance only rises to 1.06 times the initial value. 

The above results indicate that the printed film possesses excellent flexibility and thermoelectric performance compared to many previous reports (Table 1). It is worth noting that the literature reports in Table 1 do not test the entire thickness of these films. The samples are mostly prepared on substrates (such as paper or fabric) using certain methods such as spin-coating, filtration, photolithography, or cold pressing to fabricate flexible thermoelectric materials. Thus, their thickness should be similar to that of the silver selenide/paper in this study, thus allowing for a comparison of their flexibility. In order to further determine the difference in electrical performance of the film under bending and flat states, another sample is affixed to an automatic bending machine with tape, having a bending radius of 5 mm. The resistance is tested in real time as the film is bent and flattened. Throughout the approximately 350 min testing process, this printed film is bent 5000 times, collecting over 21,000 data points. As can be seen from Figure 4f, the resistance ratio (R/R_0_) remains essentially unchanged during this process, fully meeting the requirements for wearables and adapting to the complex geometric curves of the human body. 

To further investigate the flexibility of printed films, the strain (ε) associated with various radii can be determined using Equation (1): [38]

ε = t/2r × 100%(1)

where t represents the film thickness (120 μm) and r represents the radius of curvature. The strains of the film under bending are 0.4%, 0.55%, 0.67%, 0.86%, and 1.2%, respectively, corresponding to the bending radii of 15 mm, 11 mm, 9 mm, 7 mm, and 5 mm. As is widely known, the strain of inorganic semiconductors usually remains below 0.1–0.2% at room temperature [39,40]. Therefore, to further explore the reasons for the excellent flexibility of the film, we conducted a detailed characterization of the morphology of the interface under bending conditions. Under liquid nitrogen conditions, the cross-section of the film is cut with a scalpel. After the sample is processed, it remains bent for gold sputtering, followed by SEM scanning. It should be noted that the sample has been bent for at least 20 min prior to photography. Although partial fracture and delamination occur in the paper substrate under liquid nitrogen conditions, as shown in Figure S4, there is no fracture between the upper pattern and the substrate in the bent state, indicating a good adhesion between the upper pattern and the substrate. This can explain why the electrical performance of the film is nearly the same in both the bent and flat states. To accurately assess the mechanical damage to the sample following the bending test, we employ digital image correlation (DIC) technology to analyze the strain evolution of the films after a specific number of bending cycles. DIC, a technique commonly employed in both engineering and scientific fields, was utilized for the calculation of strains. In the bending experiment, we capture images of the curved portion of the film using an optical microscope after relaxation and subsequently utilize the VIC-2D system to calculate the strain of the flexible film. The experimental findings reveal that despite repeated bending, the strain increase is minimal, generally less than 0.18% (Figure S5). This implies that the specimens experienced minimal damage during the bending test.

In summary, the excellent flexibility of this printed film can be attributed to the following reasons. First, the strong bonding between the upper pattern and the flexible substrate helps the sample withstand greater strains [41,42]. This is because strain localization can lead to the rupture of the film. Once a neck forms on the film, further deformation will be concentrated in the neck, eventually causing the film to crack. Conversely, if the film is well bonded to the flexible substrate, the substrate will inhibit strain localization, which helps the film deform uniformly under high strain [41]. Paper is a flexible substrate, and the above experiments also prove that the upper pattern bonds well with the lower substrate. Additionally, the resins are good binders [28,32]. These resins, combined with heat treatment, can help the nanowires connect tightly, forming a dense film that contributes to high conductivity and flexibility [29,31,32]. Overall, compared with previous reports on flexible thermoelectric samples, this printed film demonstrates excellent flexibility. This offers a strategy for fabricating flexible TE devices using inexpensive and abundant elements.

**Table 1:** Comparison of flexible and thermoelectric parameters in flexible thermoelectric materials.

Materials	(R − R_0_)/R_0_ × 100%	Bending Radius (mm)	Bending Times	α (μV/K)	PF (μW/m K^2^)	Reference
Cu_2_Se/PI	8%	10	1000	100	500	[43]
Ag_2_Se/nylon	25%	4	1500	−140.7	1448.1	[19]
Ag_2_Te/paper	25%	5	500	−142	192	[44]
Bi_2_Te_3_/paper	140%	/	400	/	/	[45]
Ag_2_Te NWs	30%	1.5	1000	−154.96	359.76	[46]
Bi_2_Te_3_/PEDOT	4%	3.5	100	180	1350	[15]
Ag_2_Se/paper	4%	5	1000	−161	3250.9	This study
Ag_2_Se/Paper	6%	5	5000	/	/	This study

### 3.4. Stability of Printed Film

It is crucial for the device to maintain stability during its operation. In practical applications, as the samples can be influenced by air and humidity, we design experiments to assess the stability of the unsealed films. First, the film is immersed in deionized water. Then, it is taken out at regular intervals, and its resistance is measured after being wiped clean. The results reveal that after 60 min of immersion, the resistance only increased by 10% (Figure S6a). In the subsequent experiment, the samples are placed in air with a relative humidity of approximately 75%, and the resistance is measured every few days. Within 60 days, the resistance variation remains below 5% (Figure S6b). Therefore, the stability evaluation of the samples in both air and water demonstrates their potential for application in complex environments.

We conducted further reliability testing on the samples using the THB test. The THB (Temperature and Humidity Bias) test, also known as the “double 85” experiment, involves placing samples in a climate chamber at 85 °C and 85% relative humidity. The aim is to accelerate the aging process of materials and components, simulating the wear and failures that might be encountered in real-world conditions. In this study, three unencapsulated samples were tested for 7 days, with their resistance remaining virtually unchanged; the R/R_0_ values were 1.01, 1.06, and 0.98, respectively. These results suggest that the samples demonstrate outstanding stability.

## 4. Conclusions

The proposed strategy in this study is simple, environmentally friendly, and cost-effective. This approach allows for large-scale fabrication of film patterns of different shapes without the need for techniques such as photolithography, vapor deposition, or etching. The elements composing this printed film are abundantly available on Earth (the Ag and Se elements). Furthermore, this film exhibits excellent flexibility and thermoelectric performance, making it one of the best among reported flexible thermoelectric films in terms of bending resistance. Even after 5000 bends at a radius of 5 mm, its real-time resistance remains virtually unchanged. The excellent flexibility is mainly due to the paper substrate’s inhibition of strain localization in the film and the tight connection between the nanowires. In conclusion, this simple and eco-friendly strategy can be used to produce patternable, ultra-flexible thermoelectric films.

## Data Availability

The raw data supporting the conclusions of this article will be made available by the authors on request.

## Supplementary Materials

The following supporting information can be downloaded at: https://www.mdpi.com/article/10.3390/ma17153784/s1, Figure S1: The morphology of the Se NWs. (a) Scanning electron microscopy (SEM) image and (b) high-resolution TEM (HRTEM) images of the NWs. (c) XRD of the Se NWs. Figure S2: The XPS spectra of the Ag_2_Se NWs. (a) XPS survey spectrum (b) Ag 3d spectrum and (c) Se 3d spectrum. Figure S3: EDS analysis of silver selenide nanowires. Figure S4: Cross-sectional SEM images of the film under bending state. Figure S5: The printed film strains with the number of bends. (a) 0 times (b) 2000 times (c) 4000 times and (d) 5000 times. Figure S6: Electrical stability of the unpackaged device. (a) Soaked in deionized water for 60 min. (b) Exposed to air for 60 d.
