# Supplementary material for: A Strategy for Fabricating Ultra-Flexible Thermoelectric Films Using Ag2Se-Based Ink
Source: Materials (Basel). 2024 Aug 1;17(15):3784. doi: 10.3390/ma17153784 (PMC11312965; doi:10.3390/ma17153784)
Supplement: Supplementary file 1 [file materials-17-03784-s001.zip › materials-3109066-supplementary.pdf]

Supplementary Information

A strategy for fabricating ultra-flexible thermoelectric films using Ag_2Se-based ink

Yunhuan Yuan†[1], Chaogang Ding†[2], Rui Yin[1], Shun Lu[4], Jie Xu*[2], Wei Ren*[3], Kang Li*[1], Weiwei Zhao[1,2]

[1]Flexible Printed Electronics Technology Center, Harbin Institute of Technology, Shenzhen 518055, People's Republic of China. Email: likanghit@hit.edu.cn

[2]Key Laboratory of Micro-systems and Micro-structures Manufacturing of Ministry of Education, Harbin Institute of Technology, Harbin 150001, People's Republic of China. Email: xjhit@hit.edu.cn (J.X.)

[3]Department of Applied Physics, School of Physics and Electronics, Hunan University, Changsha 410082, People's Republic of China. Email: weiren@hnu.edu.cn

[4]Chongqing Institute of Green and Intelligent Technology, Chinese Academy of Sciences, Chongqing 400714, China

†These authors contributed equally

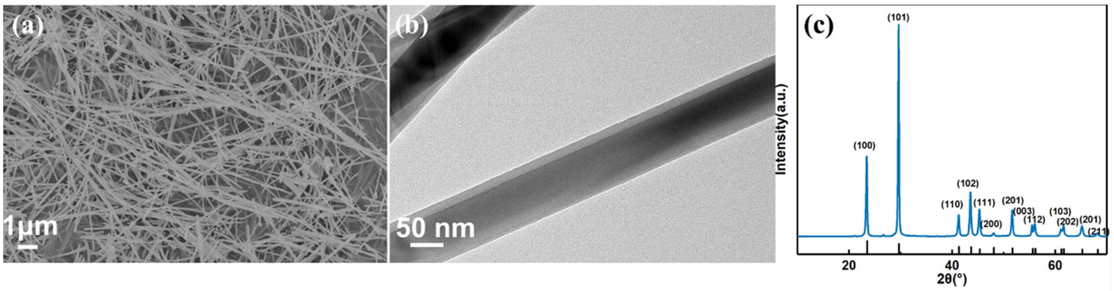

Figure S1 The morphology of the Se NWs. (a) Scanning electron microscopy (SEM) image and (b) high-resolution TEM (HRTEM) images of the NWs. (c) XRD of the Se NWs.

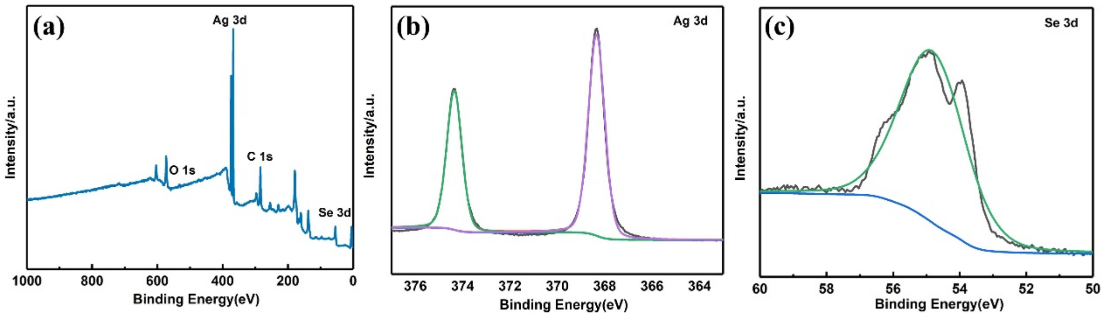

Figure S2 The XPS spectra of the Ag_2Se NWs. (a) XPS survey spectrum (b) Ag 3d spectrum and (c) Se 3d spectrum.

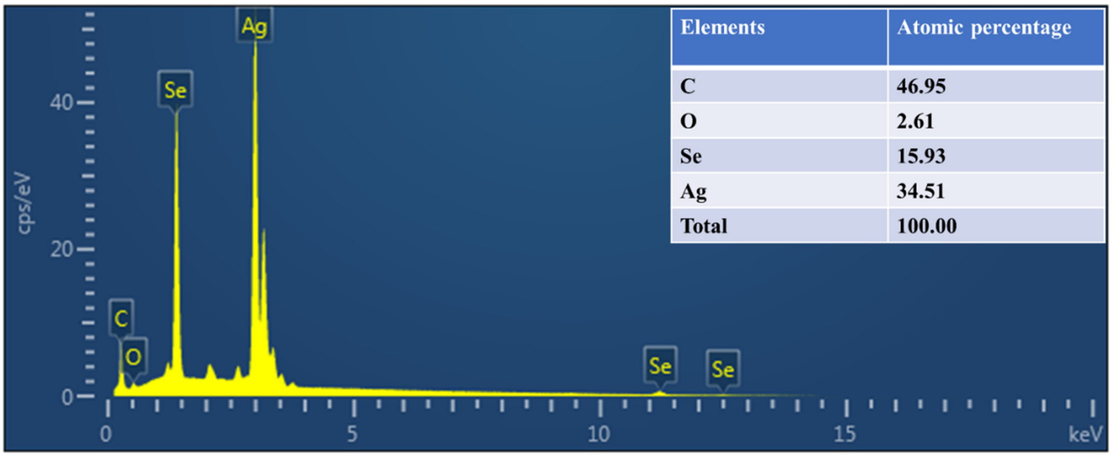

Figure S3 EDS analysis of silver selenide nanowires.

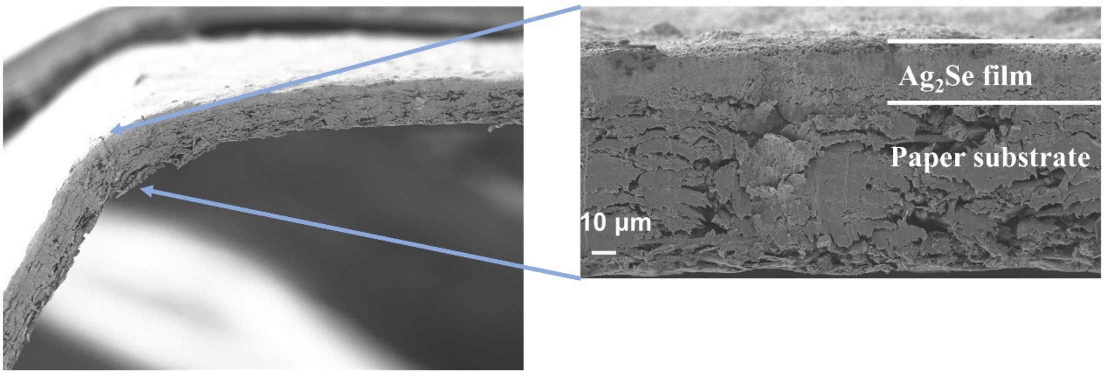

Figure S4 Cross-sectional SEM images of the film under bending state.

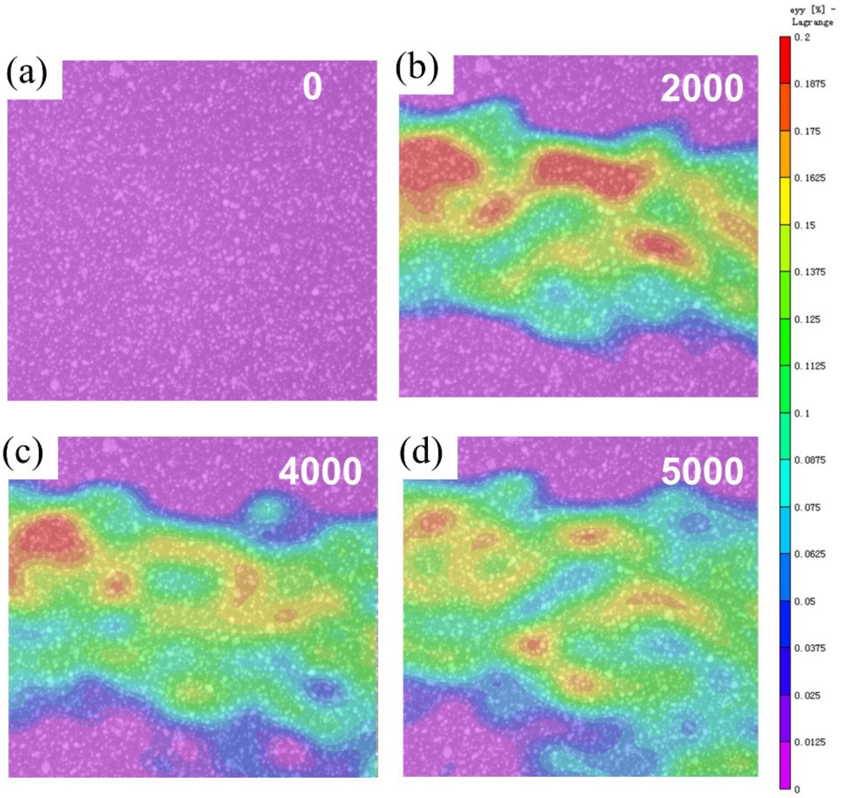

Figure S5 The printed film strains with the number of bends. (a) 0 times (b) 2000 times (c) 4000 times and (d) 5000 times.

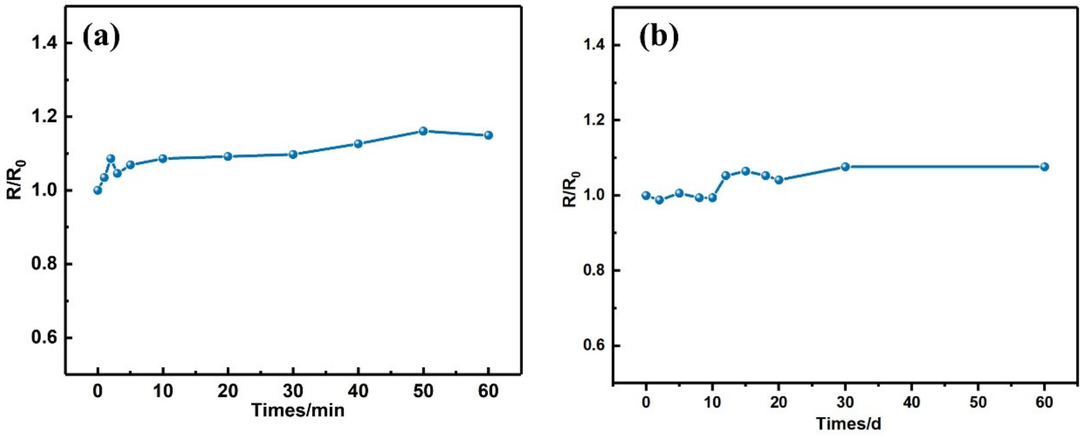

Figure S6 Electrical stability of the unpackaged device. (a) Soaked in deionized water for 60 min. (b) Exposed to air for 60 d.
